# Deep Learning for Predicting Late-Onset Breast Cancer Metastasis: The Single-Hyperparameter Grid Search (SHGS) Strategy for Meta-Tuning a Deep Feed-Forward Neural Network

**DOI:** 10.3390/bioengineering12111214

**Published:** 2025-11-07

**Authors:** Yijun Zhou, Om Arora-Jain, Xia Jiang

**Affiliations:** Department of Biomedical Informatics, University of Pittsburgh, Pittsburgh, PA 15260, USA

**Keywords:** deep learning, machine learning, meta-tunning, grid search, deep feed-forward neural network, hyperparameter tuning, breast cancer metastasis, prediction

## Abstract

**Background**: While machine learning has advanced in medicine, its widespread use in clinical applications, especially in predicting breast cancer metastasis, is still limited. We have been dedicated to constructing a deep feed-forward neural network (DFNN) model to predict breast cancer metastasis n years in advance. However, the challenge lies in efficiently identifying optimal hyperparameter values through grid search, given the constraints of time and resources. Issues such as the infinite possibilities for continuous hyperparameters like L1 and L2, as well as the time-consuming and costly process, further complicate the task. **Methods**: To address these challenges, we developed the Single-Hyperparameter Grid Search (SHGS) strategy, serving as a preselection method before grid search. Our experiments with SHGS applied to DFNN models for breast cancer metastasis prediction focused on analyzing eight target hyperparameters (epochs, batch size, dropout, L1, L2, learning rate, decay, and momentum). **Results**: We created three figures, each depicting the experimental results obtained from three LSM-I-10+-year datasets. These figures illustrate the relationship between model performance and the target hyperparameter values. Our experiments achieved maximum test AUC scores of 0.770, 0.762, and 0.886 for the 10-year, 12-year, and 15-year datasets, respectively. For each hyperparameter, we analyzed whether changes in this hyperparameter would affect model performance, examined whether there were specific patterns, and explored how to choose values for the hyperparameter. **Conclusions**: Our experimental findings reveal that the optimal value of a hyperparameter is not only dependent on the dataset but is also significantly influenced by the settings of other hyperparameters. Additionally, our experiments suggest a reduced range of values for a target hyperparameter, which may be helpful for “low-budget” grid search. This approach serves as a foundation for the subsequent use of grid search to enhance model performance.

## 1. Introduction

Machine learning (ML) has always been an important research topic in AI, and we have seen successful cases of using AI-based learning techniques such as deep learning to conduct various tasks and solve a wide range of problems in the biomedical domain. Machine learning and deep learning methods have been used in applications such as predicting drug–drug interactions using real world data collected via large-scale projects [1], modeling miRNA–mRNA interactions that cause phenotypic abnormalities in breast cancer patients [2], prioritizing disease-causing genes using sequence-based feature candidates [3,4,5], and predicting ubiquitination sites using physicochemical properties of protein sequence data [6,7]. Recent studies have also demonstrated the growing role of ML in diverse medical prediction tasks. For example, radiomics-based models have been used to identify specific breast cancer subtypes, helping to create more individualized approaches to diagnosis and therapy [8], Bayesian and machine learning models have been applied to predict how long heart surgery patients will stay in the hospital, helping hospitals plan resources and make treatment decisions [9], and bio-inspired algorithms have performed well in detecting Type 2 diabetes [10]. Furthermore, various learning methods have also been developed and applied to cancer-related prediction, such as predicting local breast cancer recurrence using language processing, identifying risk factors of prostate cancer recurrence using high-dimensional gene and clinical data [11], and predicting acute lymphoblastic leukemia (ALL) relapse in children [12].

An artificial neural network (ANN) is a machine learning framework that is designed to recognize patterns using a model loosely resembling the human brain [13,14]. Deep neural networks (DNNs), called deep learning, refer to the use of neural networks composed of more than one hidden layer [15,16,17]. DNNs have obtained significant success in commercial applications such as voice and pattern recognition, computer vision, and image processing [18,19,20,21]. However, their power has not been fully explored or demonstrated in clinical applications, such as the prediction of breast cancer metastasis, which is in part due to modeling challenges that result from the sheer magnitude of the number of variables involved in these problems [22].

**Figure 1:**
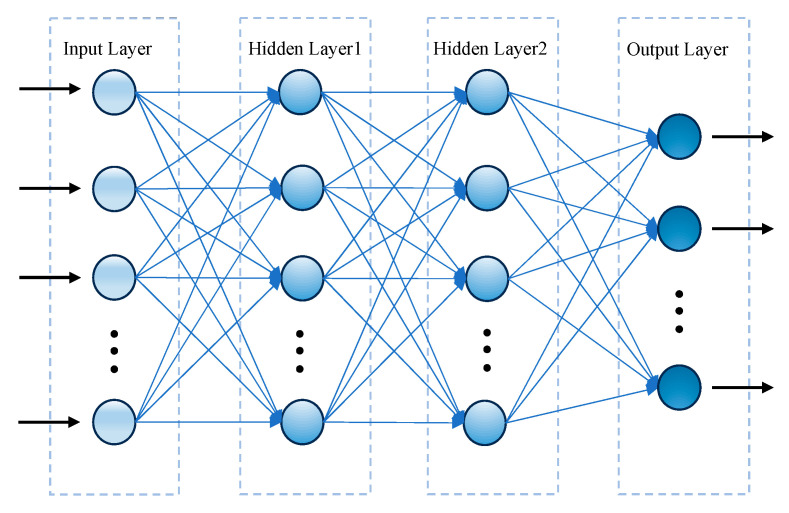
The structure of an example DFNN model that contains two hidden layers. The arrows indicate the direction of information flow from one layer to the next in the network. The three vertically arranged dots represent additional neurons omitted for clarity.

We have previously developed deep feed-forward neural network (DFNN) models that are able to predict n-year breast cancer metastasis [23]. Figure 1 illustrates the structure and the inner connections of a DFNN that we have developed. It is a four-layer neural network that contains one input layer, two hidden layers, and one output layer. The input nodes to this neural network represent the clinical features contained in the input patient data, which we also refer to as predictors, and the output layer contains one node representing the binary status of n-year breast cancer metastasis, which is also referred to as the target variable in this context.

One of the challenges of deep learning is that there are many hyperparameters that must be tuned to obtain good prediction models [24]. In deep learning, hyperparameters are the variables that determine the model’s architecture and directly influence the training process and output model performance [25]. Hyperparameters are predetermined prior to the initiation of the training cycle, and they remain constant, withstanding any learning or modification throughout the progression of the training process [24]. Tuning hyperparameters for a given dataset is a repetitive process of identifying an optimal set of hyperparameter values that produce good prediction results. We call one value assignment to the set of hyperparameters of the DFNN models a *hyperparameter setting* [23]. Grid search is a systematic way to tune hyperparameters in which each of a predetermined set of hyperparameter settings is used to train models and all trained models are compared to identify the best-performing model [23]. We previously conducted grid searches to optimize the prediction performance of our DFNN models [26]. During the grid searches, DFNN models were trained and tested corresponding to each of the preselected hyperparameter settings one at time and the average training and testing results per setting were saved.

Other approaches have been proposed to address the above-described challenge of grid search’s inefficiency. For instance, random search improves efficiency by exploring a random subset of hyperparameter combinations, achieving broader coverage while keeping computational demands low [27]. Bayesian optimization guides the search toward promising regions of the hyperparameter space using probability models, reducing the number of trails needed. Although both methods generally outperform grid search in terms of optimization speed, their primary aim is to identify the optimal combination of hyperparameters.

In contrast to these optimization-focused methods, the proposed Single-Hyperparameter Grid Search (SHGS) strategy has a different primary goal. Its focus is on systematically narrowing the search space by separating and evaluating the effect of individual hyperparameters under random background conditions. Rather than replacing existing optimization methods, SHGS complements existing methods by providing a clearer view of hyperparameter behavior, narrowing the search to more promising areas, and later fine-tuning with grid search, random search, or Bayesian optimization.

The grid searches take as an input a finite number of preselected values for each of the tunable hyperparameters. Some DFNN hyperparameters are continuous, making it difficult to predefine a suitable finite set of candidate values, so grid search can become time-consuming and computationally expensive as the number of settings increases. With a low-budget grid search for which the available computation time and resources are limited, the number of values that each hyperparameter can take is very small, often making it a difficult task to preselect the input hyperparameter settings, especially for a hyperparameter that has a very large or even an infinite number of possible values [26].

During the preselection of the input hyperparameter settings, we often face unanswered or under-answered questions, such as: will changing the values of a particular hyperparameter truly have a significant effect on model performance? How does model performance change as the value of a hyperparameter increases, and are there particular patterns? If there are performance patterns associated with a hyperparameter, would they be dataset-dependent? When selecting a hyperparameter value, should we concentrate on lower values, mid-range values, or higher values? With a low-budget grid search, what would be a rule of thumb for selecting a promising set of values for a hyperparameter that can take a very large number of values?

The primary motivation of this study is to address this challenge of hyperparameter preselection, which often constrains model optimization and reproducibility. To this end, we introduce the SHGS strategy, which we describe in detail in the Section 2 below. The main goals of this work are as follows: (1) to provide an efficient pre-search approach that guides the selection of promising hyperparameter ranges; (2) to analyze the sensitivity of model performance to specific hyperparameters; and (3) to offer empirical insights for tuning DFNN models in clinical prediction tasks. We conducted experiments using the SHGS with our DFNN models for predicting late-onset breast cancer metastasis, which we define as metastasis that occurred at least 10 years after the initial treatment. We identified eight target hyperparameters that have a large or an infinite number of values (epochs, batch size, dropout, L1, L2, learning rate, decay, and momentum).

## 2. Methods

### 2.1. The SHGS Framework

The purpose of running an SHGS is to look for a promising but reduced range of values for a particular hyperparameter, which we call the target hyperparameter in the SHGS, to assist in the task of preselecting input values for the subsequent grid search. In a SHGS, we will first give a large range of values for the target hyperparameter that has an infinite number of possible values, such as L1 and L2, while all the other hyperparameters will each take only one preselected value. So, an SHGS can be considered as a special type of grid search in which the number of hyperparameter settings used for model training during the grid search is equal to the number of values of the target hyperparameter. In this study, we identified 8 hyperparameters that can take an infinite number of values and treated them as our target hyperparameters. These 8 hyperparameter are epochs, batch size, learning rate, dropout rate, momentum, decay, L1 weight decay, and L2 weight decay. Table 1 shows a description of the 8 target hyperparameters and their values that we studied via our SHGS experiments. In these experiments, to avoid bias, the fixed value used by each of the non-target hyperparameters was pre-picked randomly from the pool of all values considered for each of these hyperparameters following a uniform distribution. We call one value assignment of these non-target hyperparameters a background hyperparameter setting. Table 1 also shows all values of the non-target hyperparameters that are considered. For each of the target hyperparameters, we repeated the SHGS experiment 10 times and each time used a different randomly selected background hyperparameter setting from all possible settings in order to see how a different background setting can affect the results. Repeating each experiment 10 times helped to reveal consistent hyperparameter effects while mitigating randomness from varying background settings and balancing stability with computational cost. We used Python along with the TensorFlow and Keras libraries to implement SHGS, which is now available at [https://pypi.org/project/SHGS], accessed on 31 October 2025.

### 2.2. Experiments

**Figure 2:**
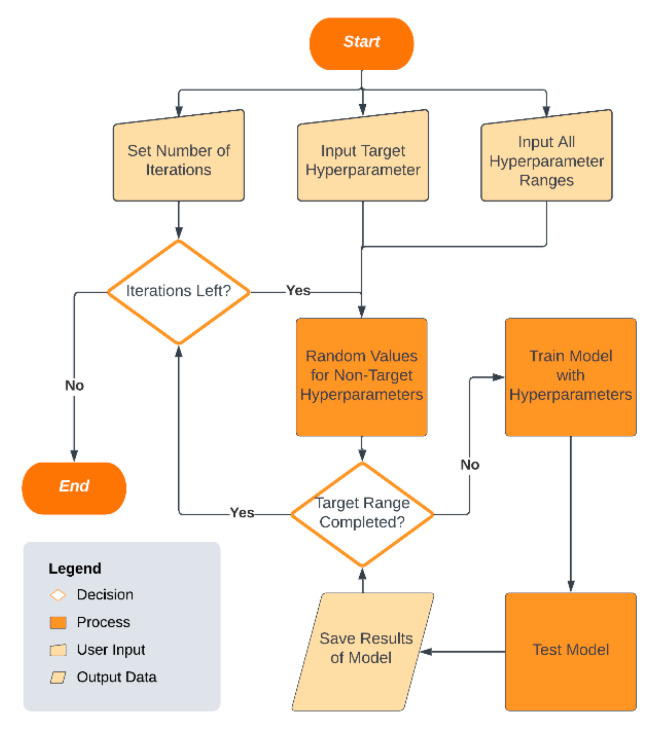
A flowchart illustrating the proposed SHGS strategy and its experimental procedure.

Figure 2 presents the workflow of the proposed SHGS framework, summarizing its key components (iteration control, random background hyperparameter selection, model training, and result recording). The SHGS framework takes three main inputs (the number of iterations, the target hyperparameter, and the value ranges for all hyperparameters). The number of iterations determines how many times the SHGS process is executed for a given target hyperparameter. In each iteration, random values are assigned to all non-target hyperparameters to form a background hyperparameter setting. For the target hyperparameter, each value within its specified range is systematically explored in conjunction with the background setting. The SHGS algorithm then sequentially updates the target hyperparameter, performs 5-fold cross-validation (see details below) to train models under each target hyperparameter setting, tests the resulting model on an independent test dataset, and saves the outcomes.

### 2.3. The Evaluation of Model Performance Using Five-Fold Cross-Validation

For a given binary diagnostic test, a receiver operator characteristic (ROC) curve plots the true positive rate against the false positive rate for all possible cutoff values [28]. The area under an ROC curve (AUC) measures the discrimination performance of a model [29]. We followed a 5-fold cross-validation (CV) procedure to train and evaluate models under each hyperparameter setting in an SHGS. The dataset was first split into two subsets (an 80% training dataset for optimizing the model’s performance and a separate 20% independent test dataset to evaluate its generalization ability). The entire training dataset was then divided evenly into 5 sub-datasets. The division was mostly conducted randomly, and, in the meantime, we made sure that each sub-dataset had approximately 20% of the positive cases and 20% of the negative cases to guarantee an overall balanced distribution of the two. For each target hyperparameter setting, we conducted both the training and testing five times. Each time, a model was learned from a different set of 4 sub-datasets combined, and then it was tested using the remaining sub-datasets as the validation set. The average training and testing AUC across all five times, which are called mean_train_auc and mean_test_auc, respectively, were reported via the grid search package. Additionally, to evaluate the model’s performance completely independently, we used the 20% set-aside test dataset to test the output model refitted using the entire training dataset under each target hyperparameter setting to obtain a test_auc.

While the mean test AUC from cross-validation was used as an internal validation metric, the test AUC obtained from the independent hold-out set was adopted as the final evaluation metric to analyze performance trends across a wide range of target hyperparameter values—the primary goal of our exploratory study. All experiments were executed using the same computing environment to ensure consistent runtimes and performance evaluations. Each grid search and SHGS experiment was conducted on a shared pool of machines, with each job allocated 16 CPU cores and 64 GB of RAM. No GPU acceleration was used, and early stopping was deliberately disabled to allow for full observation of convergence behavior. The implementation was developed in Python using the Keras (v2.4.3) and Scikit-Learn (v0.24.2) packages. All runs were executed under identical software configurations to ensure reproducibility. We used this procedure for all datasets involved in this research. Given the class imbalance in our datasets and the need to compare many hyperparameter settings efficiently, AUC was chosen as the primary evaluation metric due to its robustness, threshold-independence, and interpretability in imbalanced settings.

### 2.4. Datasets

In this research, we used three datasets, LSM-I-10Year, LSM-I-12Year, and LSM-I-15Year, which include breast cancer patients with at least 10, 12, and 15 years of follow-up, respectively. These datasets were extracted directly from the broader LSM-I-nYear dataset based on follow-up duration, which had already undergone data cleaning, missing value handling, and feature selection in our prior study [30]. As such, no additional preprocessing was necessary in the current work.

The LSM-I-nYear dataset was originally constructed in our previous study [31] using clinical records from the Lynn Sage Database (LSDB) and the Northwestern Medicine Enterprise Data Warehouse (NMEDW) at Northwestern Medicine. In that study, we applied the Model-Based Interaction Learning (MBIL) method to identify predictors whose interactions are strongly associated with the disease outcome, such as metastasis in this study’s context. This method, first proposed in our earlier work [30], is based on Bayesian network modeling and information-theoretic criteria and is designed to discover predictive feature interactions relevant to clinical outcomes. Table 2 below shows the counts of the cases and predictors included in the three datasets. A description of these predictors is included in Supplementary Tables S1–S3 and a previously published paper [23].

## 3. Results

**Figure 3:**
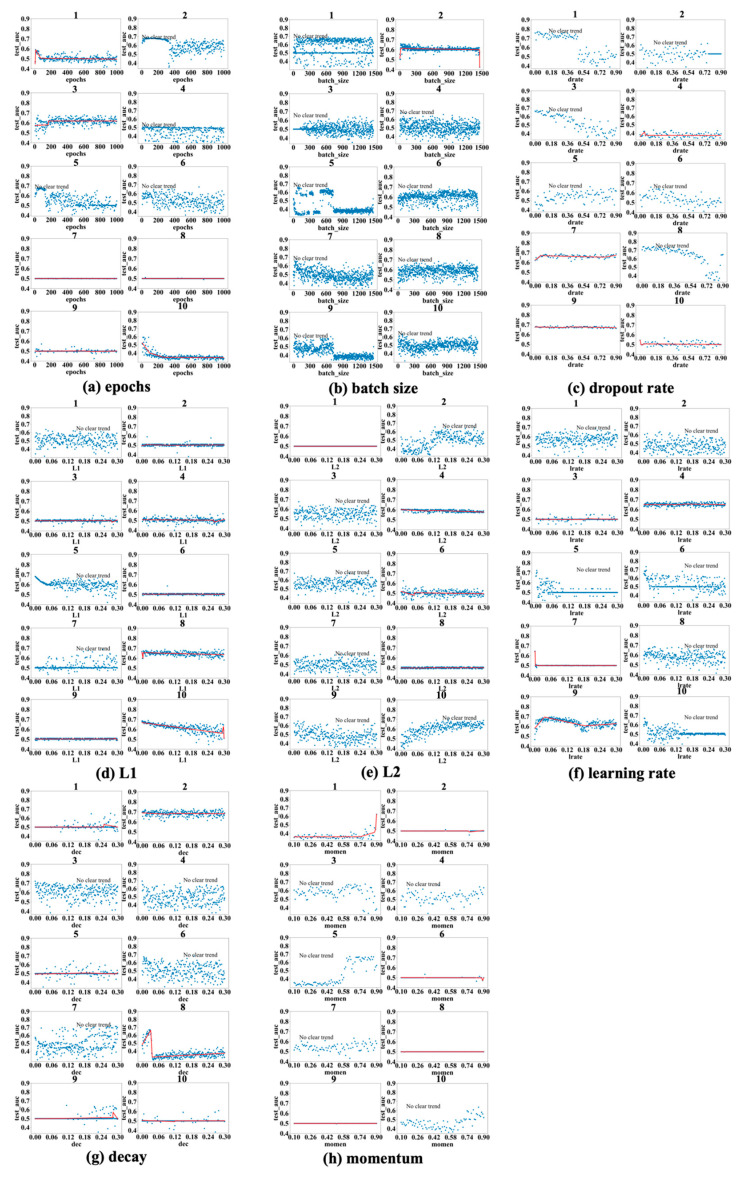
Scatter plots: test_auc vs. a target hyperparameter concerning LSM-I-10Year. Each panel (**a**–**h**) corresponds to one of the eight target hyperparameters examined in the SHGS experiments. Within each panel, the ten numbered subplots (1–10) represent results from ten independent SHGS runs, each conducted under a distinct random background hyperparameter configuration. The red line represents the piecewise linear regression fit illustrating the trend of model performance across the hyperparameter range.

**Figure 4:**
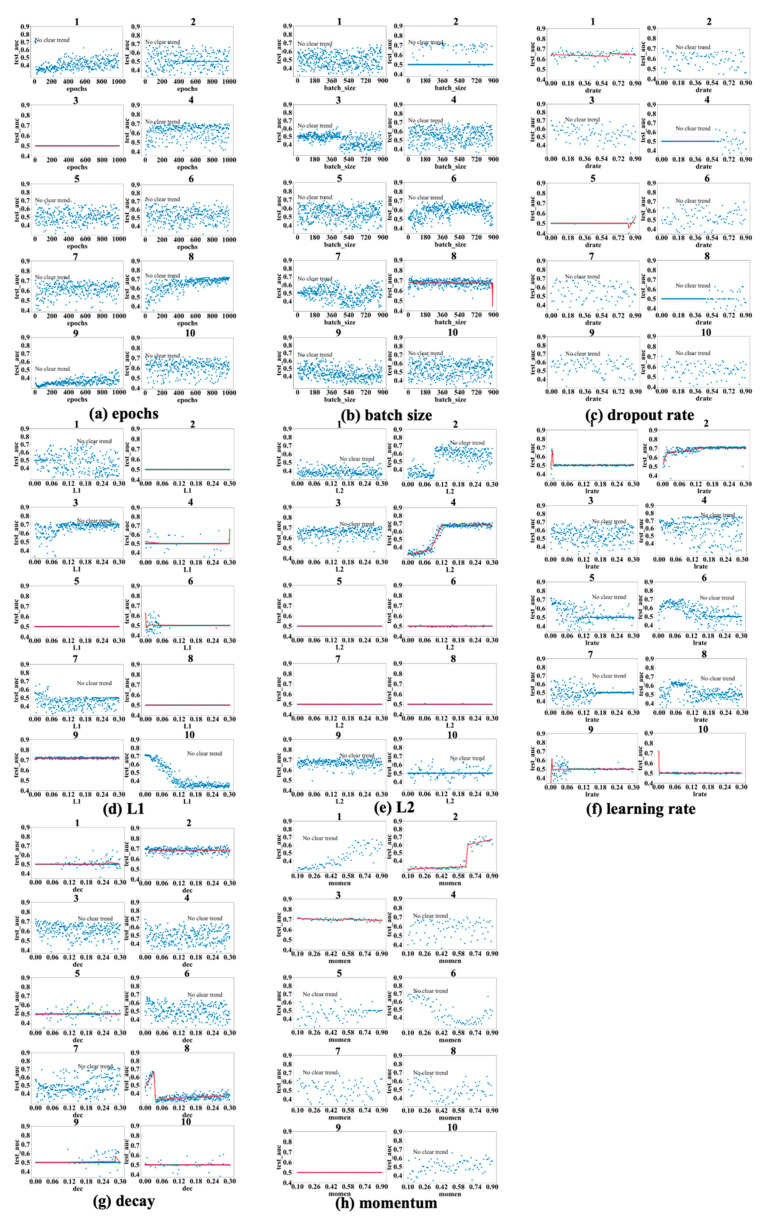
Scatter plots: test_auc vs. a target hyperparameter concerning LSM-I-12Year. Plot elements are defined as in Figure 3.

**Figure 5:**
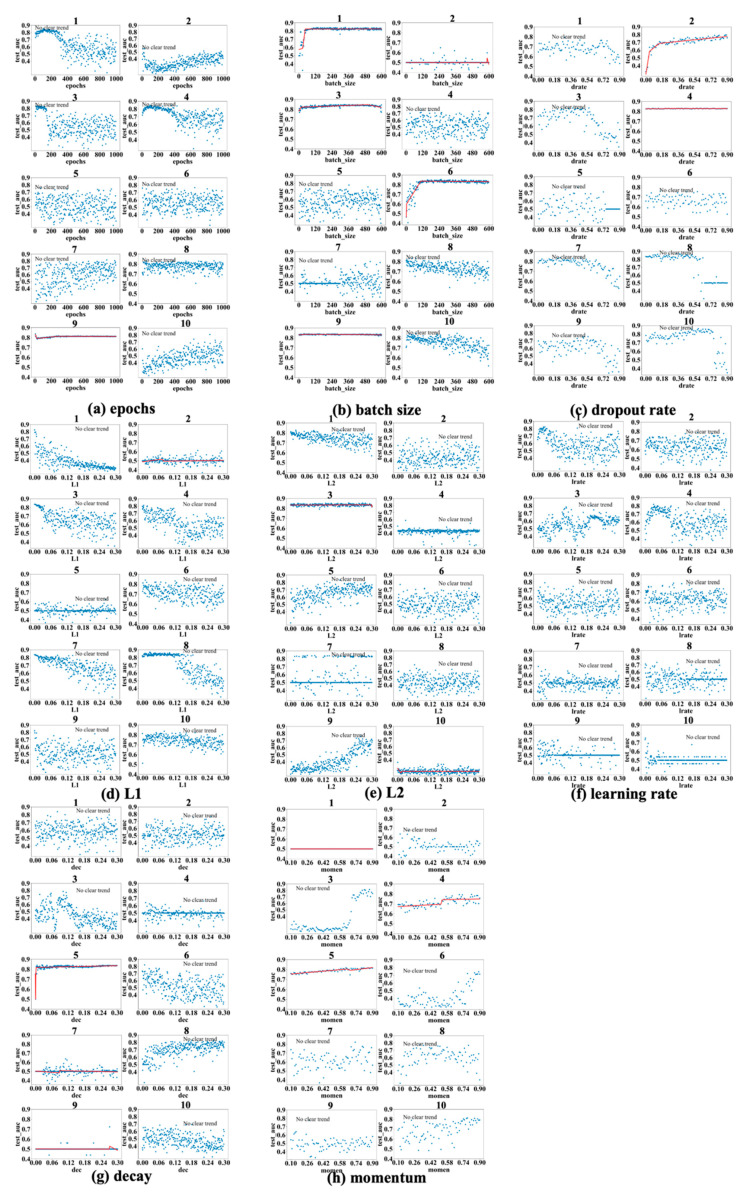
Scatter plots: test_auc vs. a target hyperparameter concerning LSM-I-15Year. Plot elements are defined as in Figure 3.

Figure 3, Figure 4 and Figure 5 show the results obtained for the LSM-I-10Year, LSM-I-12Year, and LSM-I-15Year datasets, respectively. Each figure consists of eight panels of scatter plots, one for each of the eight target hyperparameters. Within each panel are ten individual scatter plots, each showing the results of a specific SHGS experiment conducted using the corresponding dataset for the corresponding target hyperparameter. Such a scatter plot demonstrates how model performance, as measured by test_auc, changes when the values of a target hyperparameter increase during an SHGS experiment.

We analyzed performance trends using three-segment piecewise linear regression fitted with the pwlf package, which effectively captures segmented relationships in hyperparameter spaces. To reduce noise, trend lines were displayed only when the mean squared error (MSE) between the fitted curve and observed test AUC values was below 0.001; otherwise, the figure was labeled as “no clear trend”. Interestingly, some hyperparameters exhibit clear trends in one dataset but not in others, suggesting that the effectiveness of a hyperparameter is influenced by the background setting. Conversely, a few hyperparameters demonstrate consistent trends across all three datasets, indicating they may exert a more robust influence on model performance.

Based on the results shown in Figure 3, Figure 4 and Figure 5, the scatter plots of the 10 different experiments for the same target hyperparameter are quite different. This is perhaps because each experiment used a different background setting that was randomly selected by the SHGS scheme. Note that, with certain hyperparameter configurations that were randomly generated via grid search, the prediction performance was very poor, indicated by the consistently low test_AUC values (approximately 0.5), as seen in figures such as Figure 3a experiments 7 and 8. An AUC value of 0.5 indicates that the model is unable to distinguish between positive and negative class points, performing no better than random guessing [32].

The contrast between successful runs (e.g., Figure 3a(5)), which reveal clear performance trends, and failed runs (e.g., Figure 3a(7,8)), where performance remains near chance level (AUC ≈ 0.5), reveals that SHGS results are highly dependent on the background hyperparameter configurations used. Successful runs occur when the background configuration provides a stable setting for optimization, allowing the effect of the target hyperparameter to be observed clearly. In contrast, failed runs arise when suboptimal background combinations disrupt the optimization process, obscuring the true influence of the target hyperparameter and illustrating how background variability can affect the reliability of SHGS results.

As to epochs (Figure 3a, Figure 4a and Figure 5a), the highest test_auc values for the 10-year, 12-year, and 15-year data are 0.683 (Figure 3a(5)), 0.727 (Figure 4a(8)), and 0.852 (Figure 5a(1)), respectively. We notice that optimal test_auc values are often reached at low epochs (e.g., Figure 3a(5) and Figure 5a(1)). In addition, we see the following three different patterns: (1) test_auc decreases once the number of epochs surpasses a specific threshold (e.g., Figure 3a(2) and Figure 5a(3)), (2) test_auc plateaus after passing a certain point (e.g., Figure 4a(8)) or throughout (e.g., Figure 5a(9)), and (3) test_auc steadily goes up as the number of epochs passes a certain point (e.g., Figure 4a(1) and Figure 5a(10). We also notice that, in some cases, test_auc has high variance and fluctuates rapidly while the number of epochs increases (e.g., Figure 4a(6)), while it has very low variance in some other cases (e.g., Figure 5a(9)).

In the batch size experiments (Figure 3b, Figure 4b and Figure 5b), the best test_auc values are 0.7 (Figure 3b(6)) for the 10-year dataset, 0.738 (Figure 4b(8)) for the 12-year dataset, and 0.856 (Figure 5b(6)) for the 15-year dataset. There tends to be a short warm-up period (batch size < 50) when performance is unstable but reaches a peak quickly, and, in most experiments and over all three datasets, performance reaches the peak before the batch size increases to one-fifth of its highest value. Overall, we see the following three patterns after performance reaches its peak: (1) a slight negative correlation between performance and batch size (Figure 3b(7), Figure 4b(9) and Figure 5b(8,10)); (2) a sharp dip when the batch size reaches about half of its highest value, but the trend remains stable at either side of the sharp dipping point (Figure 3b(5,9), Figure 4b(3,7) and Figure 5b(7)); and (3) the trend remains constant, with or without large variance, and this is seen in most cases, indicating that a larger batch size tends to have a limited influence on performance improvement.

Figure 3c, Figure 4c and Figure 5c show the patterns in dropout rate. The highest scores are 0.77 (10-year dataset), 0.726 (12-year dataset), and 0.886 (15-year dataset). Similar to batch size, in most cases, the performance trend remains constant or becomes worse after it quickly reaches a peak. The performance tends to become very poor after the dropout passes 0.5, with some exceptions (e.g., Figure 3c(5), Figure 4c(1) and Figure 5c(4)).

Regarding the L1 and L2 regularization parameters, denoted as L1 and L2, respectively, in subsequent discussions, the best test_auc values for L1 in Figure 3d, Figure 4d and Figure 5d are 0.689, 0.734, and 0.872, and the best test_auc results for L2 in Figure 3e, Figure 4e and Figure 5e are 0.706, 0.734, and 0.86. The results of L1 reveal no increasing trend in performance as measured by test_auc when L1 values increase in any of the 10 experiments. Based on these results, using a small L1 value (<0.03) in grid search can be sufficient to obtain the best-performing model. The L2 results demonstrate similar trends in most cases with some exceptions, in which it requires a larger L2 value to reach the performance peak in experiments (Figure 3e(2,10), Figure 4e(2,4) and Figure 5e(9)), but none of these experiments gave the best test_auc values.

The highest test_auc scores for learning rate (Figure 3f, Figure 4f and Figure 5f) are 0.734 (Figure 3f(6)), 0.762 (Figure 4f(4)), and 0.832 (Figure 5f(1)), respectively. The best test_auc values in most experiments were obtained when the learning rate was below 0.03. After reaching the peak, the performance tends to stabilize (e.g., Figure 4f(2)), or, alternatively, it exhibits a significant decline with increased fluctuations after passing a certain point (e.g., Figure 5f(4)).

The best test_auc values achieved for decay are 0.706 (Figure 3g(10) for the 10-year dataset), 0.733 (Figure 4g(2) for the 12-year dataset), and 0.87 (Figure 5g(4) for the 15-year dataset). All experiments that achieved the best test_auc values reached the best performance almost immediately and the increase in the decay rate had no significant impact on performance. Certain patterns are seen for other experiments, such as the eighth experiment of Figure 4g, where the test_auc value initially rises as the decay rate increases but drops abruptly at a point (around 0.03) and remains suboptimal. Overall, the performance reaches the peak when the decay rate assumes a small value (below 0.03), then the trend either stays constant or goes down as the decay rate increases, with the exceptions seen in Figure 5g(8,10), where it takes longer for the performance to reach the peak.

For momentum, the best test_auc values are 0.675 (Figure 3h(3) for the 10-year dataset), 0.72 (Figure 4h(6) for the 12-year dataset), and 0.835 (Figure 5h(3) for the 15-year dataset). Considering all three datasets, the performance trend demonstrates two patterns (stays constant or goes up after momentum at least passes the half-way point), but the well-performing models tend to appear with the second pattern. For example, for the 10-year data, the performance reaches the peak after momentum passes 0.5 in the experiment in which we identified the best model (Figure 3h(3)), and, for the 15-year data, we obtained the best model after momentum passed 0.7. For the 12-year dataset, the best test_auc was obtained when momentum was below 0.3 and the trend after the peak test_auc went down as an exception, but we obtained near-best models in other experiments when the momentum passed 0.5 (Figure 4h(1)) and when it passed 0.7 (Figure 4h(2)).

**Figure 6:**
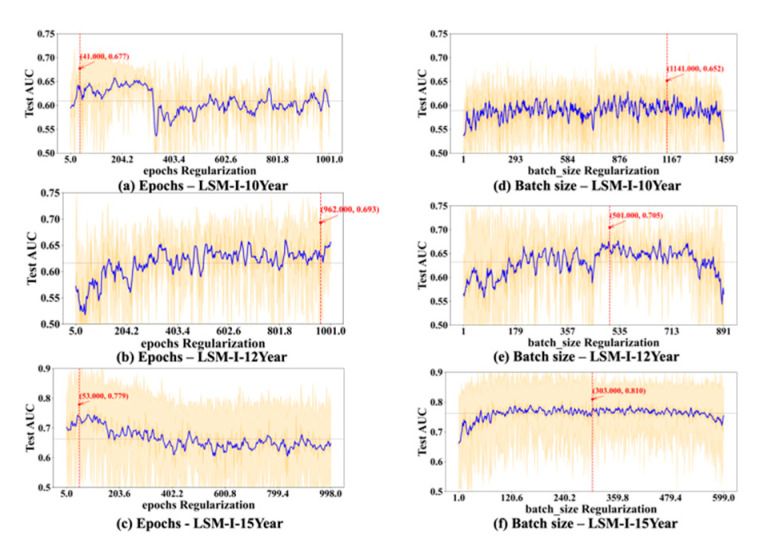
Impact of epochs and batch size on test AUC. The blue line represents the smoothed performance trend across the ten SHGS experiments, the shaded area indicates variance across trials, and the red marker denotes the highest observed AUC and its corresponding hyperparameter value.

**Figure 7:**
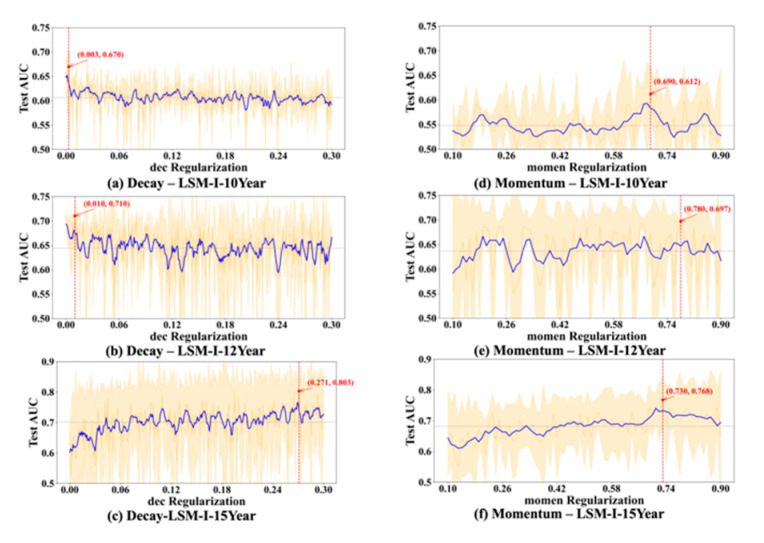
Impact of decay and momentum on test AUC. Plot elements are defined as in Figure 6.

**Figure 8:**
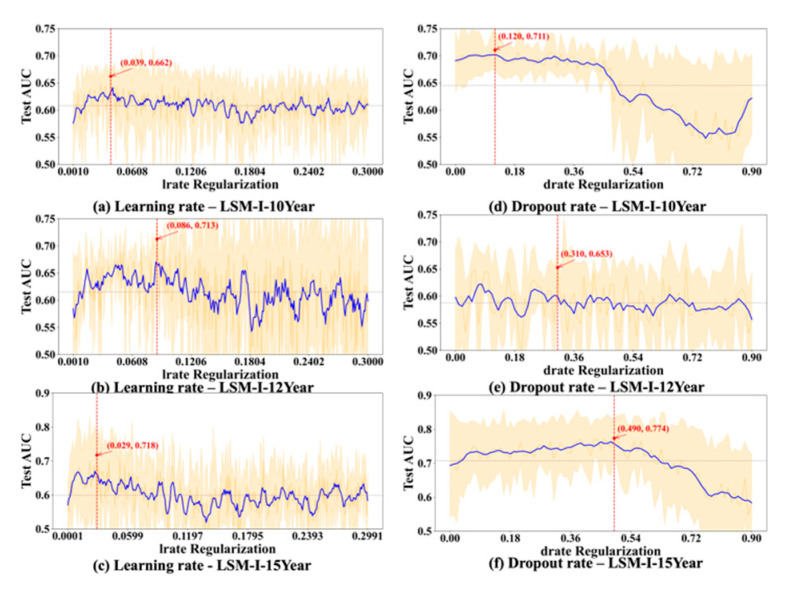
Impact of learning rate and dropout rate on test AUC. Plot elements are defined as in Figure 6.

**Figure 9:**
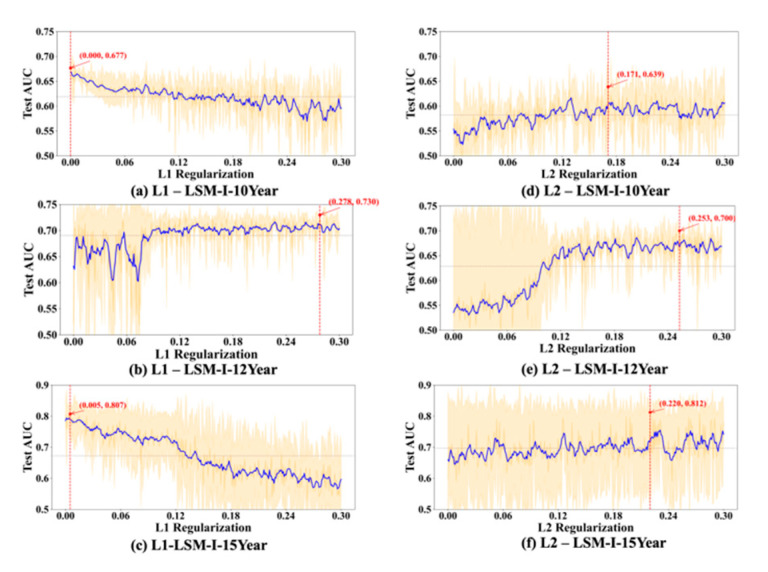
Impact of L1 and L2 on test AUC. Plot elements are defined as in Figure 6.

To further evaluate the stability and reliability of our experimental results, we conducted a statistical analysis on the performance of each hyperparameter. For every value of the tested hyperparameter, we computed the mean and standard deviation of the AUC across multiple trials. As shown in Figure 6, Figure 7, Figure 8 and Figure 9, the blue curve represents the smoothed performance trend, while the shaded area indicates the variance across experiments. To focus on valid configurations, we excluded models with AUC < 0.55, which are typically caused by ineffective random parameter combinations. Figure 6, Figure 7, Figure 8 and Figure 9 omit low-performing cases (shown in Figure 3, Figure 4 and Figure 5) to better reveal optimal hyperparameters, performance trends, and stability, as including the numerous failed runs shown in Figure 3, Figure 4 and Figure 5 would obscure the underlying meaningful patterns. In addition, the red marker denotes the highest AUC observed across all 10 experiments, along with its corresponding hyperparameter value.

Based on Figure 6, Figure 7, Figure 8 and Figure 9, we observed several hyperparameters exhibiting consistent performance trends across all three datasets, including batch size, momentum, L2 regularization, learning rate, and dropout rate, while others—specifically the number of training epochs, weight decay, and L1 regularization—demonstrated dataset-specific effects. As shown in Figure 6d–f, a medium-to-small batch size consistently yielded superior AUC performance. Similarly, high momentum values were beneficial across all datasets (Figure 7d–f), supporting accelerated convergence. Learning rate exhibited a consistent preference for smaller values in the range of 0.01–0.08 across datasets (Figure 8a–c). Likewise, lower dropout rates (0.05–0.2) outperformed higher rates consistently (Figure 8d–f). L2 regularization was broadly effective across datasets (Figure 9d–f), with the AUC generally improving as L2 increased, though the trend often plateaued beyond a moderate value.

In contrast, certain hyperparameters required dataset-specific tuning. The optimal number of training epochs varied; both the LSM-I-10Year and LSM-I-15Year datasets favored fewer epochs, likely to prevent overfitting, whereas the LSM-I-12Year dataset benefited from extended training. Weight decay exhibited a similar divergence, with the 10Year and 12Year datasets performing better under weaker regularization, while the 15Year dataset achieved improved performance with stronger decay. For L1 regularization, both the 10Year and 15Year datasets preferred minimal or no L1 penalty, indicating that feature sparsity was not beneficial. In contrast, the 12Year dataset showed marginal performance gains with increased L1 strength.

Table 3 displays the number of hyperparameter settings and corresponding running times for the SHGS experiments conducted using the three LSM-I datasets. Table 4 below represents the total experimental time for each of the eight target hyperparameters studied across the same datasets. Surprisingly, the experiments targeting L2 as the hyperparameter exhibit the longest duration among all three datasets.

**Figure 10:**
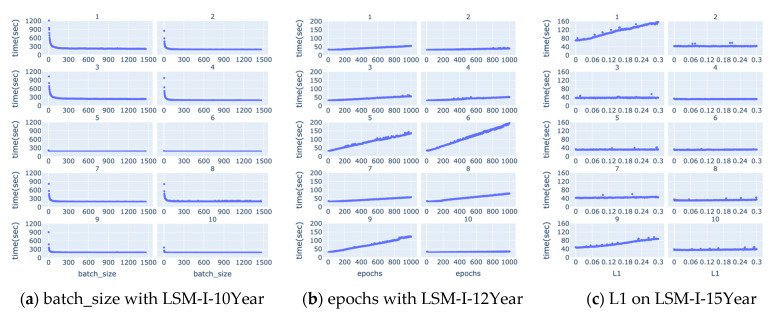
Selected scatter plots: running time vs. a target hyperparameter. (**a**) Batch size with LSM-I-10Year, (**b**) Epochs with LSM-I-12Year, and (**c**) L1 regularization with LSM-I-15Year. The blue line represents the smoothed trend of running time across the hyperparameter range.

We also studied how running time changes along with each of the hyperparameters, but, as shown in Supplementary Figures S1–S3, there are no apparent correlations between running time and the target hyperparameter in most cases. There are a few notable correlations between the running time and target hyperparameters such as batch size, epochs, and L1 as shown in Figure 10 below. Based on Figure 10a, running time decreases quickly as the value of batch size increases till it reaches a turning point, from which the running time either remains unchanged or decreases very slowly and slightly. The turning point often occurs when the batch size is very small, mostly below 50. Figure 10b shows that there is often an apparent positive correlation between the running time and epochs. Figure 10c demonstrates that occasionally there is a positive correlation between the running time and L1, but, in most experiments, increasing the value of L1 does not affect the running time much.

These patterns align with the design of the SHGS framework, which separates one target hyperparameter while randomizing others to expose how each hyperparameter independently contributes to model performance under diverse background settings. Furthermore, the piecewise regression analysis and Figure 6, Figure 7, Figure 8 and Figure 9 quantitatively show that SHGS captures non-linear and segmented response patterns in hyperparameter–performance relationships, identifying appropriate value ranges and highlighting the stability or context-dependence of each parameter. The runtime analysis (Table 3 and Table 4, Figure 10) demonstrates SHGS’s efficiency, providing comparable insights at a fraction of the cost of a full grid search, consistent with its role as a preselection step for meta-tuning.

## 4. Discussion

Previous studies reported that the best model performance may occur at either very high epochs [33] or at very low values (below 10) [34] depending on dataset characteristics [35]. Our findings refine this understanding by not only confirming the dataset-dependent nature of optimal epoch values (Figure 6a–c) but also revealing that model performance is substantially influenced by background hyperparameter settings. As shown in Figure 3a, the effects of epochs can vary dramatically across different background configurations, even within the same dataset.

In addition, our results (Figure 10b) show that running time is often correlated with the number of epochs, so using a high number of epochs could significantly increase the computation time, which renders it a lesser choice especially when the time available to run a grid search is limited. These observations indicate the importance of adaptive training schedules. Instead of statically assigning epoch values across the board, future systems could benefit from early stopping mechanisms or dynamic adjustment strategies based on convergence signals or overfitting risk [35,36].

The optimal batch size remains an open question, with prior studies reporting conflicting findings. Some suggest that smaller batch sizes tend to yield improved results [37], while others observed a higher test accuracy with larger batches [38]. This inconsistency may arise from differences in datasets and background hyperparameter configurations. Additionally, performance often peaks when the batch size is less than half the dataset size (Figure 6d–f), supporting claims that large batches may converge to sharp minima and impair generalization [36,39]. As such, constraining batch size values to the lower range (e.g., <50% of the maximum tested value) may serve as an effective strategy for low-budget grid searches, particularly when computational resources or time are limited [40].

Finding a dropout rate that works perfectly for all datasets and situations is difficult, as it is influenced by various factors [37,38,41,42]. The dataset size seems to be a key factor. Research indicates that smaller dropout rates are beneficial for small datasets [43] and remain effective for large datasets. Our results show that the performance trend mostly remains constant or goes down after it reaches the peak, which often occurs before the dropout rate reaches 0.5. This substantiated that a dropout rate of *p* = 0.5 works well for various networks as suggested in [22]. Combining our experimental results and the previous findings, using a dropout rate of 0.5 or a few values that are less than or equal to 0.5 might be a good initial choice for a low-budget grid search.

L1 regularization is suggested to be more effective than L2 regularization and dropout [44,45,46]. The authors of [44] suggest that an L1 value of 0.001 is preferable when compared with 0.0001, 0.01, and 0.1. According to [45], if the L1 value is too high, the model is too simple, leading to underfitting, while if it is too low, the model becomes too complex and may overfit. Based on our experimental results, regardless of the dataset, the result is consistently better when using a smaller L1 value, and the optimal test_auc values were consistently achieved when L1 was less than 0.03. L2 regularization was more uniformly beneficial, with performance improving as the penalty increased up to a point. Several experiments (e.g., Figure 3e(2,10), Figure 4e(2,4) and Figure 5e(4)) achieved the peak AUC at relatively large L2 values (>0.03), although these settings did not produce the highest test AUCs overall. Therefore, for low-budget grid search settings, a narrow L1 range near zero (e.g., 0–0.01) is advisable, while a broader L2 range (e.g., 0–0.3) may be necessary to capture the regularization benefit before performance declines.

Selecting an appropriate learning rate for grid search is nontrivial, as it must balance convergence speed and stability—being large enough to ensure efficiency, yet small enough to avoid overshooting or oscillation [47,48]. In each of our experiments, we tested a large range of learning rate values (0 to 0.3), but, across all three datasets, the best test_auc values most often occurred before the learning rate reached 0.03. We also noticed that once test_auc reached a peak, it rarely went up as the learning rate further increased. These experimental results suggest that a suitable range of learning rate values for a low-budget grid search is below 0.03.

Researchers currently tend to determine the values of learning rate decay (referred to as decay in this context) based on empirical considerations, including 0.1, 0.01, and 0.001 [46]. Due to the relationship between decay and learning rate, the magnitude of the learning rate is a crucial factor to consider when determining the value of decay [46]. While many experiments achieved the peak AUC at low decay values (typically <0.03), suggesting that minimal regularization is often adequate, this pattern was not consistent across all datasets. The 15-year dataset experiments (e.g., Figure 7c) exhibited markedly better performance at high decay values (e.g., >0.5), indicating that stronger regularization can be beneficial in certain contexts. Therefore, for low-budget grid searches, decay values between 0 and 0.003 (approximately one-tenth of the upper-bound learning rate) are generally sufficient. However, when resources permit, extending the search range up to 0.8 may better accommodate dataset-specific variations.

Momentum directly influences optimization during model training and expedites convergence by incorporating historical gradients [49]. Previous research suggests that values around 0.9 seem to be good values [45,46]. Some studies suggest that the interaction between momentum and learning rate matters more than their individual values [50]. In our experiments, we evaluated 81 momentum values ranging from 0.1 to 0.9 to ensure comprehensive coverage. Results indicate that optimal model performance most frequently occurs when momentum exceeds 0.5, with two of the three highest test AUC scores obtained at values above 0.7. Nonetheless, isolated cases showed strong performance at lower momentum settings; for instance, the best result for the LSM-I-12Year dataset was achieved with momentum below 0.3. Taken together, our findings suggest that a momentum range of 0.7 to 0.9 is a strong initial choice for grid search. When computational resources allow, exploring lower momentum values (e.g., 0.01–0.03) may further uncover dataset-specific optima. These findings also highlight a key limitation of a fully randomized design: some hyperparameters, such as momentum, only take effect under specific conditions (e.g., with an SGD-type optimizer). This reinforces that a hyperparameter’s relevance and influence are inherently context-dependent.

An unexpected but notable finding from our experiments was the significantly longer runtime observed for L2 regularization. Because L1 regularization did not exhibit a similar slowdown, this cannot be explained by a simple computational overhead. We hypothesize that this behavior reflects an interaction effect between the L2 penalty and certain unfavorable background hyperparameter configurations. Unlike L1, which promotes sparsity by pushing many weights to exactly zero and can simplify the effective network structure, L2 applies a continuous constraint on all weights. This universal pressure, when combined with a poor background setting—such as a high learning rate or an unstable weight initializer—could create highly unstable gradient dynamics. Consequently, the optimizer may be forced to perform substantially more internal computations at each step to manage these conflicting update signals, leading to a prolonged runtime. This result highlights that hyperparameter interactions may affect not only performance outcomes but also computational efficiency—an aspect often overlooked in practice.

## 5. Conclusions

We examined how eight key hyperparameters affect the prediction of late-onset breast cancer metastasis, aiming to define efficient search ranges under limited computational resources. Our results show that optimal hyperparameter values vary across datasets and depend on the configuration of other parameters, referred to here as the background setting. Despite this variability, we provide practical guidance for conducting low-budget grid searches in similar tasks.

We propose the following reduced value ranges as a strong initial choice: epochs, fewer than 100; batch size, less than half the number of data points; dropout rate, ≤0.5 is commonly an effective initial choice; L1, 0 to 0.03; L2, a range of 0 to 0.3 can be explored; learning rate, <0.03; decay, 0 to 0.003 is often sufficient, though extending the search up to 0.8 may be beneficial if resources permit; momentum, 0.7 to 0.9 is recommended, while broader ranges (e.g., 0.01–0.03) may be included if resources allow. It is crucial to note, however, that these recommendations were empirically derived from our specific task of predicting late-onset breast cancer metastasis using a DFNN, so they should be interpreted as task- and model-specific observations rather than universally optimal settings. Furthermore, for this kind of clinical prediction, time-to-event or survival models could offer a more realistic approach worth investigating in future studies.

Nevertheless, the SHGS framework itself is versatile and can be adapted to other prediction tasks to narrow hyperparameter ranges before performing full-scale optimization. In practice, running a few SHGS trials beforehand can help locate high-potential regions and improve overall tuning efficiency. Although this study focused on using a DFNN to predict late-onset breast cancer metastasis, the SHGS framework holds potential for broader applications. Future research may extend SHGS to other architectures, such as convolutional and recurrent neural networks, and to multimodal data (e.g., imaging, text, and omics data) in order to evaluate its generalizability across domains. Additionally, since the current SHGS relies on random background sampling, future work could develop more sophisticated sampling strategies. A promising direction is to enhance the SHGS framework with a constraint system or conditional sampling to ensure that only valid and meaningful hyperparameter combinations are explored, thereby increasing the efficiency and interpretability of the exploratory process. Finally, enhancing SHGS from its current PyPI implementation into a more comprehensive and modular open-source toolkit could further improve its usability and broaden its potential applicability in biomedical and general machine learning research.

## Figures and Tables

**Table 1:** Hyperparameters and their values used in SHGS experiments.

Hyperparameter	Description	Values Used in SHGS Experiments	Number of Values Studied per SHGS Experiment (Target Hyperparameter Only)
Epochs	Number of times the model is trained by the full training dataset	5~1001, step size 3	333
Batch size	Number of samples that are processed together in a single forward and backward pass during training	1 to the # of datapoints in a dataset	730 in LSM-10Year-I dataset 446 in LSM-12Year-I dataset 300 in LSM-15Year-I dataset
Learning rate	Control the learning and parameter update speed during optimization	0.001~0.3, Step size 0.001	300
Dropout rate	Mitigate overfitting and training time by randomly ignoring nodes	0~0.9, Step size 0.01	91
Momentum	Speed up optimization by incorporating historical gradients into parameter updates. Momentum is exclusively applicable within the SGD optimizer.	0.1~0.9, Step size 0.01	81
Decay	Iterative decay of the learning rate by applying a decreasing factor at each epoch	0~0.3, Step size 0.001	301
L1 weight	Control the strength of L1 regularization in model training	0~0.3, Step size 0.001	301
L2 weight	Control the strength of L2 regularization in model training	0~0.3, Step size 0.001	301
# of Hidden Layers	The depth of a DNN model	1,2,3,4	Non-target hyperparameter
# of Hidden Nodes	Number of neurons in a hidden layer	All integers from 1 to the number of datapoints of each dataset	Non-target hyperparameter
Optimizer	Optimizes model parameters during the training process toward minimizing the loss	SGD, Adam, Adagrad, Nadam, Adamax	Non-target hyperparameter
Weight Initializer	A technique employed to assign initial values to the weights of the connections between neurons in a neural network	Constant, Glorot_normal, Glorot_uniform, He_normal, He_uniform	Non-target hyperparameter
Input Layer Activation Function	A function applied to the input data of a neural network’s input layer	Relu, Sigmoid, Softmax, Tanh	Non-target hyperparameter
Hidden Layer Activation Function	A function applied to the output of a hidden layer in a neural network	Relu, Sigmoid, Softmax, Tanh	Non-target hyperparameter
Output Layer Activation Function	A function applied to the output of a neural network’s output layer, producing the final prediction of the network.	Sigmoid	Non-target hyperparameter
Loss Function	A method of evaluating the dissimilarity between the predicted output of a model and the actual values	Binary_crossentropy	Non-target hyperparameter

**Table 2:** Case and predictor count of the three LSM-I-10^+^-year datasets.

	Total # of Cases	# Positive Cases	# Negative Cases	#Number of Predictors
LSM-I-10year	1827	572	1255	18
LSM-I-12year	1115	588	527	20
LSM-I-15year	751	608	143	17

**Table 3:** Number of hyperparameter settings and running time per dataset in SHGS experiments.

Dataset	Number of Hyperparameter Settings	Unit Running Time per Hyperparameter Setting	Total Running Time per Dataset
LSM-I-10Year	24,380	93.875 s	635.74 h
LSM-I-12Year	21,540	71.454 s	427.532 h
LSM-I-15Year	20,080	76.849 s	428.646 h

**Table 4:** Running time (in hours) per target hyperparameter.

Target Hyperparameter	Total Running Time	Running Time per Dataset		
		LSM-I-10Year	LSM-I-12Year	LSM-I-15Year
Epochs	178.003	113.047	29.568	35.388
Batch size	145.69	77.542	59.293	8.855
L1	170.957	115.336	47.719	7.903
L2	466.878	103.453	161.839	201.586
Dropout rate	102.28	51.569	10.393	40.318
Learning rate	125.213	23.894	64.082	37.237
Momentum	107.467	85.205	5.818	16.444
Decay	195.429	65.694	48.82	80.915

## Data Availability

The original data presented in this study are openly available in datadryad.org at DOI 10.5061/dryad.64964m0 (https://datadryad.org/dataset/doi:10.5061/dryad.64964m0).

## Supplementary Materials

The following supporting information can be downloaded at: https://www.mdpi.com/article/10.3390/bioengineering12111214/s1, Figure S1: Scatter plots: running time vs. the values taken by the target hyperparameters concerning LSM-I-10Year; Figure S2: Scatter plots: running time vs. the values taken by the target hyperparameters concerning LSM-I-12Year.png; Figure S3: Scatter plots: running time vs. the values taken by the target hyperparameters concerning LSM-I-15Year; Table S1: A Description of the Variables of the LSM-10Year Dataset; Table S2: The variables of the LSM-12Year Dataset; Table S3: The variables of the LSM-15Year Dataset.
