# Supplementary material for: Deep Learning for Predicting Late-Onset Breast Cancer Metastasis: The Single-Hyperparameter Grid Search (SHGS) Strategy for Meta-Tuning a Deep Feed-Forward Neural Network
Source: Bioengineering (Basel). 2025 Nov 7;12(11):1214. doi: 10.3390/bioengineering12111214 (PMC12650627; doi:10.3390/bioengineering12111214)
Supplement: Supplementary file 1 [file bioengineering-12-01214-s001.zip › bioengineering-3894208-supplementary.pdf]

Supplement

Table S1. A Description of the Variables of the LSM-10Year Dataset

Variables included	Description	Values
ethnicity	ethnicity of patient	not Hispanic, Hispanic
smoking	smoking history of patient	ex smoker, non smoker, cigarettes, chewing tobacco, cigar
alcohol usage	alcohol usage of patient	moderate, no use, use but nos (non otherwise specified), former user, heavy user
family history	family history of cancer	cancer, no cancer, breast cancer, other cancer, cancer but nos
age_at_diagnosis	age at diagnosis of the disease	0-49, 50-69, >69
TNEG	triple negative status in terms of patient being ER, PR, and HER2 negative	yes, no
ER	estrogen receptor expression	neg, pos, low pos
ER_percent	percent of cell stain pos for ER receptors	0-20, 20-90, 90-100
PR	progesterone receptor expression	neg, pos, low pos
PR_percent	percent of cell stain pos for PR receptors	0-20, 20-90, 90-100
HER2	HER2 expression	neg, pos
n_tnm_stage	# of nearby cancerous lymph nodes	0, 1, 2, 3, 4, X
stage	composite of size and # positive nodes	0, 1, 2, 3
lymph_nodes_positive	number of positive lymph nodes	0, 1-8, >8
histology	tumor histology	lobular, duct
grade	grade of disease	1, 2, 3
DCIS_level	type of ductal carcinoma in situ	solid, apocrine, cribriform, dcis, comedo, papillary, micropapillary
surgical_margins	whether residual tumor	res. tumor, no res. tumor,<br>no primary site surgery
distant recurrence (Metastasis)	This is the target variable	yes, no

Table S2. The variables of the LSM-12Year Dataset

Variables included	Description	Values
race	race of patient	white, black, Asian, American Indian or Alaskan native, native Hawaiian or other Pacific islander
ethnicity	ethnicity of patient	not Hispanic, Hispanic
family history	family history of cancer	cancer, no cancer, breast cancer, other cancer, cancer but nos
age_at_diagnosis	age at diagnosis of the disease	0-49, 50-69, >69
menopausal_status	inferred menopausal status	pre, post
ER	estrogen receptor expression	neg, pos, low pos
ER_percent	percent of cell stain pos for ER receptors	0-20, 20-90, 90-100
PR	progesterone receptor expression	neg, pos, low pos
PR_percent	percent of cell stain pos for PR receptors	0-20, 20-90, 90-100
P53	whether P53 is mutated	neg, pos, low pos
HER2	HER2 expression	neg, pos
n_tnm_stage	# of nearby cancerous lymph nodes	0, 1, 2, 3, 4, X
stage	composite of size and # positive nodes	0, 1, 2, 3
lymph_nodes_positive	number of positive lymph nodes	0, 1-8, >8

histology	tumor histology	lobular, duct
size	size of tumor in mm	0-32, 32-70, >70
grade	grade of disease	1, 2, 3
invasive	whether tumor is invasive	yes, no
DCIS_level	type of ductal carcinoma in situ	solid, apocrine, cribriform, dcis, comedo, papillary, micropapillary
surgical_margins	whether residual tumor	res. tumor, no res. tumor, no primary site surgery
distant recurrence (Metastasis)	This is the target variable	yes, no

Table S3. The variables of the LSM-15Year Dataset

Variables included	Description	Values
race	race of patient	white, black, Asian, American Indian or Alaskan native, native Hawaiian or other Pacific islander
alcohol usage	alcohol usage of patient	moderate, no use, use but nos (non otherwise specified), former user, heavy user
age_at_diagnosis	age at diagnosis of the disease	0-49, 50-69, >69
menopausal_status	inferred menopausal status	pre, post
ER	estrogen receptor expression	neg, pos, low pos
ER_percent	percent of cell stain pos for ER receptors	0-20, 20-90, 90-100
t_tnm_stage	prime tumor stage in TNM system	0, 1, 2, 3, 4, IS, 1mic, X
n_tnm_stage	# of nearby cancerous lymph nodes	0, 1, 2, 3, 4, X
stage	composite of size and # positive nodes	0, 1, 2, 3
lymph_node_status	patient had any positive lymph nodes	neg, pos
size	size of tumor in mm	0-32, 32-70, >70
grade	grade of disease	1, 2, 3
histology2	tumor histology subtypes	IDC, DCIS, ILC, NC
invasive_tumor_location	where invasive tumor is located	mixed duct and lobular, duct, lobular, none
re_excision	removal of an additional margin of tissue	yes, no
surgical_margins	whether residual tumor	res. tumor, no res. tumor, no primary site surgery
histology	tumor histology	lobular, duct
distant recurrence (Metastasis)	This is the target variable	yes, no

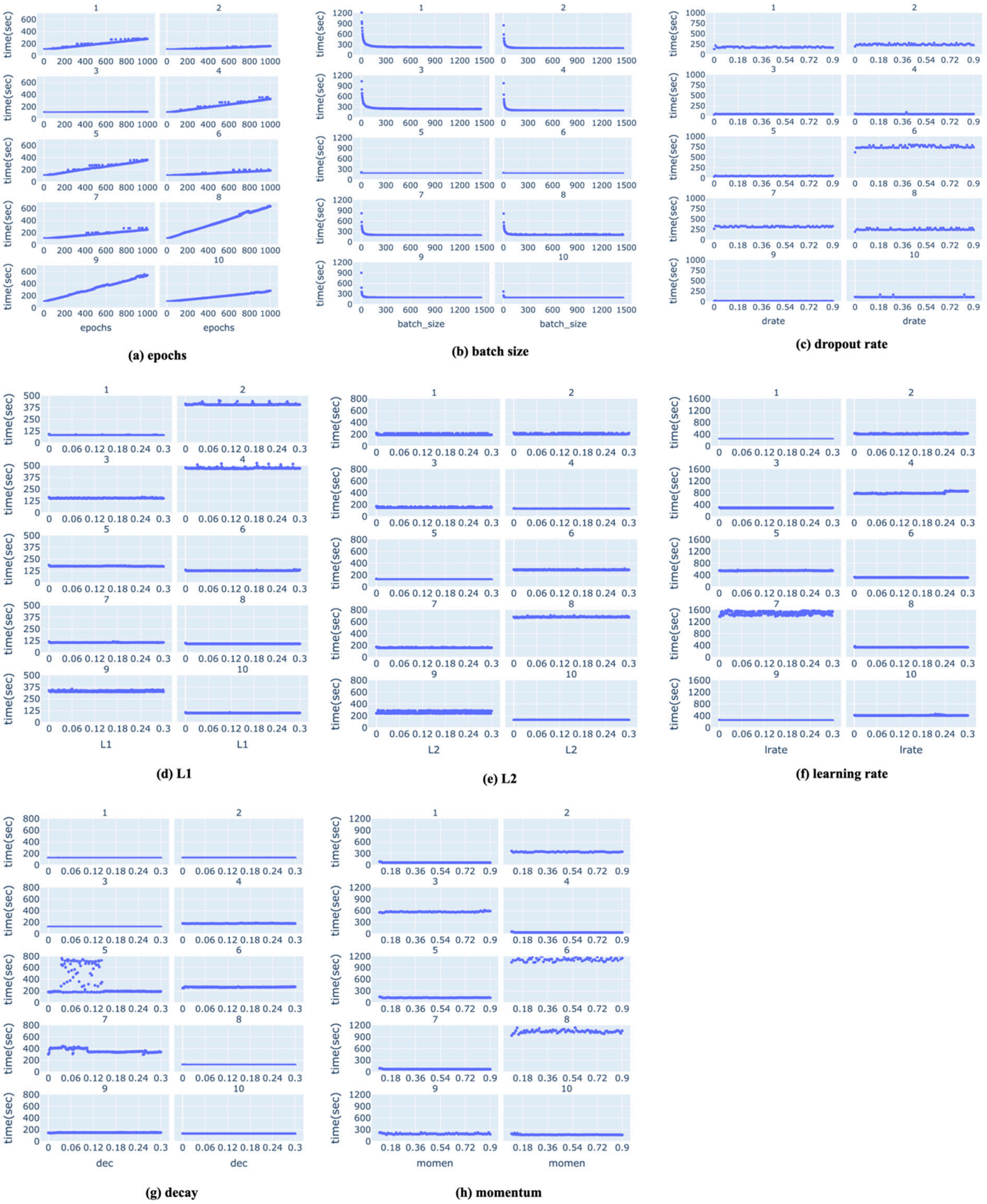

Figure S1. Scatter plots: running time vs. the values taken by the target hyperparameters concerning LSM-I-10Year

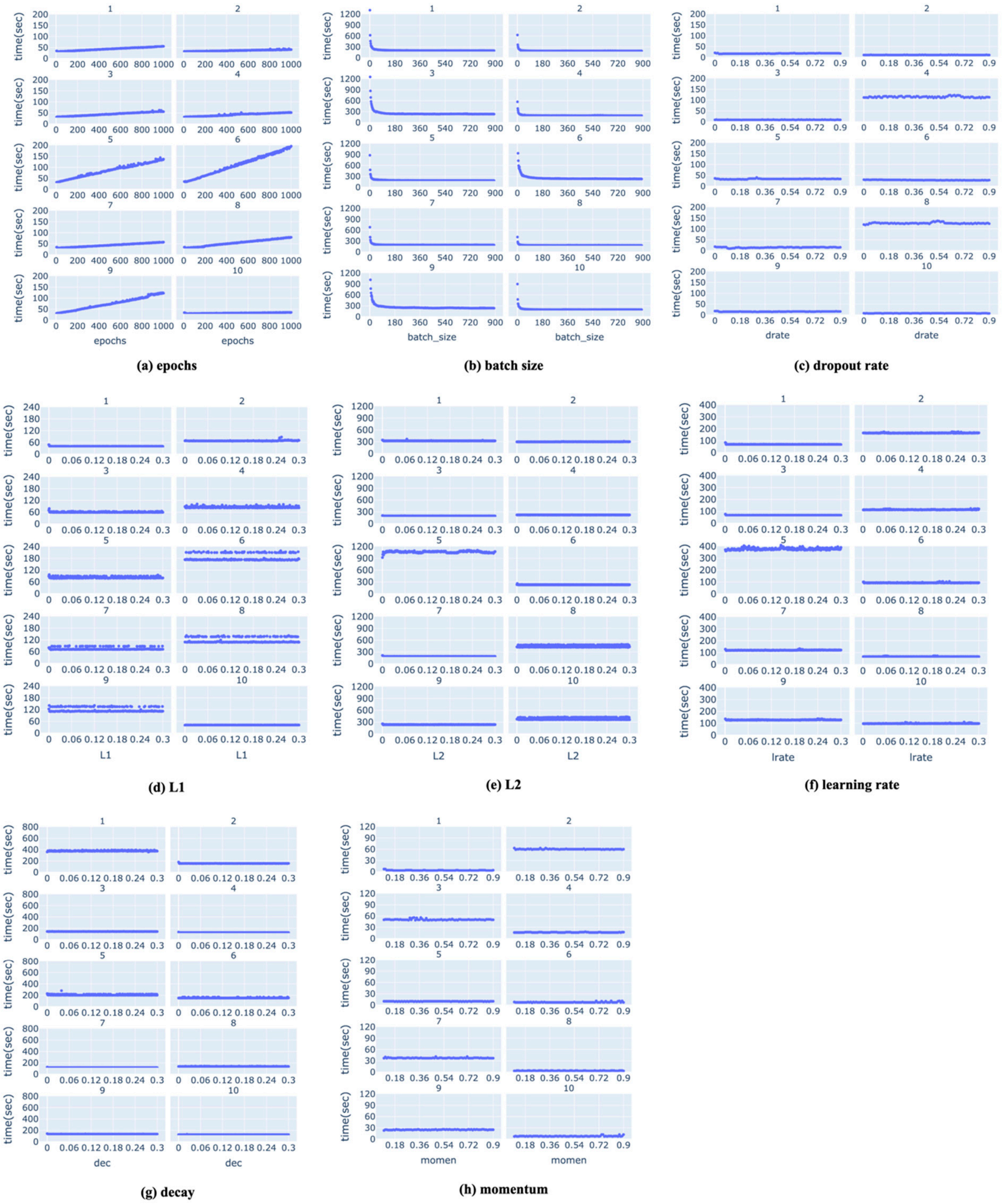

Figure S2. Scatter plots: running time vs. the values taken by the target hyperparameters concerning LSM-I-12Year.png

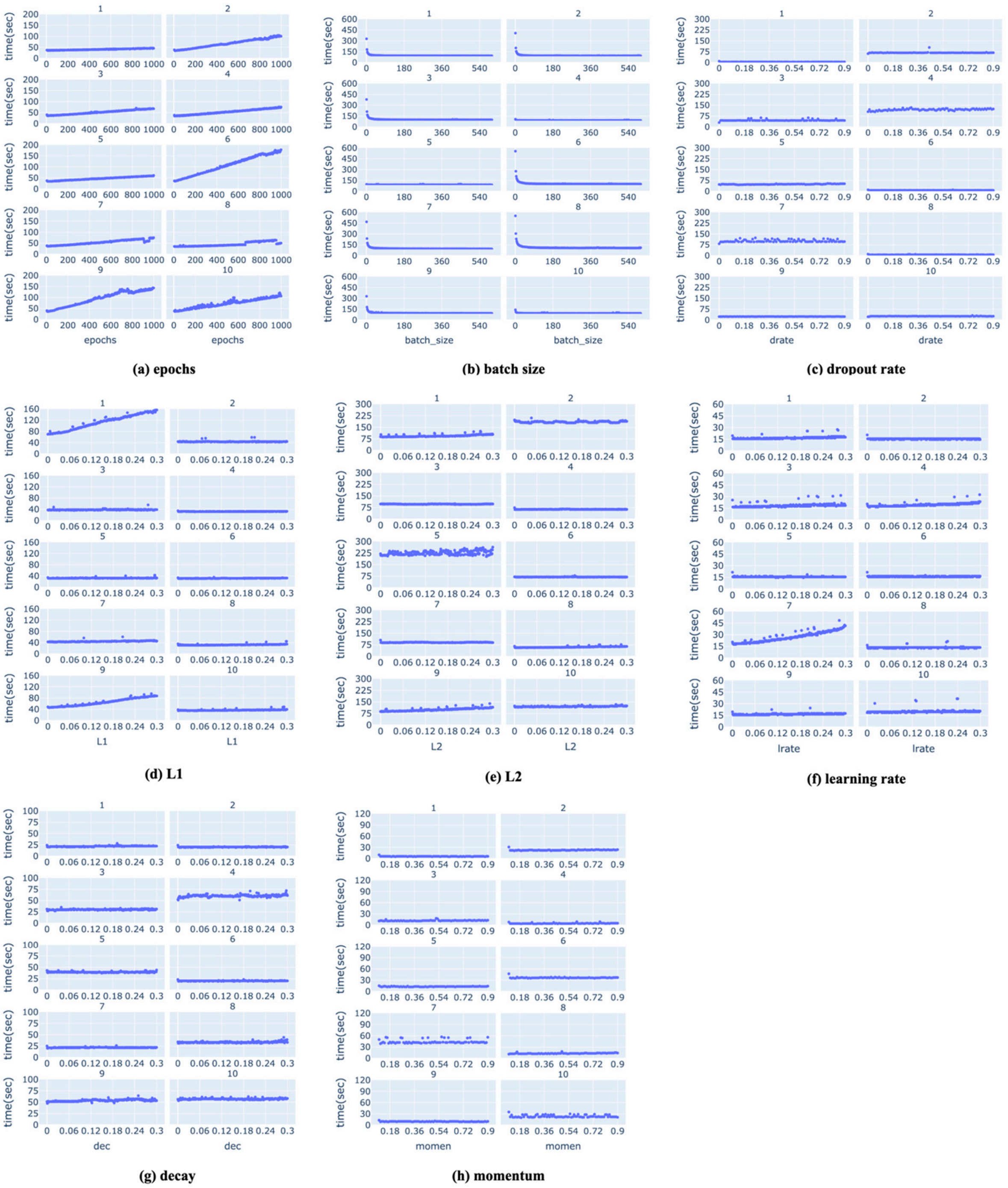

Figure S3. Scatter plots: running time vs. the values taken by the target hyperparameters concerning LSM-I-15Year
